# Mixed phenotype acute leukemia in a child associated with a *NUP98‐NSD1* fusion and *NRAS* p.Gly61Arg mutation

**DOI:** 10.1002/cnr2.1372

**Published:** 2021-03-30

**Authors:** Shireen S. Ganapathi, Sunil S. Raikar, Svetlana A. Yatsenko, Miroslav Djokic, Andrew Bukowinski

**Affiliations:** ^1^ Division of Hematology/Oncology, Department of Pediatrics Seattle Children's Hospital, University of Washington Seattle Washington USA; ^2^ Department of Pediatrics, Aflac Cancer and Blood Disorders Center Children's Healthcare of Atlanta and Emory University Atlanta Georgia USA; ^3^ Department of Pathology, UPMC Cytogenetics Laboratory University of Pittsburgh School of Medicine Pittsburgh Pennsylvania USA; ^4^ Division of Hematopathology, Department of Pathology University of Pittsburgh School of Medicine Presbyterian/Shadyside Pittsburgh Pennsylvania USA; ^5^ Division of Hematology/Oncology Children's Hospital of Pittsburgh of UPMC Pittsburgh Pennsylvania USA

**Keywords:** mixed phenotype acute leukemia (MPAL), mTOR inhibitors, *NRAS*, *NUP98‐NSD1*

## Abstract

**Background:**

Mixed phenotype acute leukemia (MPAL) is a rare subset of acute leukemia in the pediatric population associated with genetic alterations seen in both acute lymphoblastic leukemia (ALL) and acute myeloid leukemia (AML).

**Case:**

We describe a patient with MPAL with a *NUP98* (nucleoporin 98)‐*NSD1* gene fusion (nuclear receptor binding SET domain protein1) and *NRAS* (neuroblastoma RAS viral oncogene homolog mutation) p.Gly61Arg mutation who was treated with upfront AML‐based chemotherapy, received hematopoietic stem cell transplant (HSCT), but unfortunately died from relapsed disease.

**Conclusion:**

This case highlights the challenges faced in choosing treatment options in MPAL patients with complex genomics, with predominant myeloid features.

**Abbreviations**
ALL: acute lymphoblastic leukemia
AML: acute myeloid leukemia
B/My MPAL: B‐lymphoid/myeloid mixed phenotype acute leukemia
CBC: complete blood count
CML: chronic myeloid leukemia
ETP: early T‐cell precursor
FCA: flow cytometric analysis
FISH: fluorescence in situ hybridization
GvHD: graft‐vs‐host‐disease
HSCT: hematopoietic stem cell transplantation
MAC: myeloablative conditioning
MPAL: mixed phenotype acute leukemia
mTOR: mammalian target of rapamycin
NGS: next generation sequencing
*NRAS*
: neuroblastoma RAS viral oncogene homolog mutation
*NSD1*
: nuclear receptor binding SET domain protein1
*NUP98*
: nucleoporin 98
RIC: reduced intensity conditioning
T/My MPAL: T‐lymphoid/myeloid mixed phenotype acute leukemia

## 1 INTRODUCTION

Mixed phenotype acute leukemia (MPAL) is a rare subset of acute leukemia, accounting for 2% to 5% of cases and characterized by blasts of myeloid and lymphoid lineage.^1^, ^2^, ^3^ Genomic analysis reveals wide genetic heterogeneity with fusion genes or molecular genotypes associated with AML or ALL, complicating treatment decisions.^4^, ^5^, ^6^ We present the case of a 13‐year‐old female with MPAL with a *NUP98*‐*NSD1* gene fusion and a *NRAS* p.Gly61Arg mutation. The patient received upfront AML therapy followed by hematopoietic stem cell transplantation (HSCT), and relapsed shortly after HSCT, but showed a partial response to the combination of sirolimus and azacitidine before succumbing to her disease.

## 2 CASE DESCRIPTION

**FIGURE 1:**
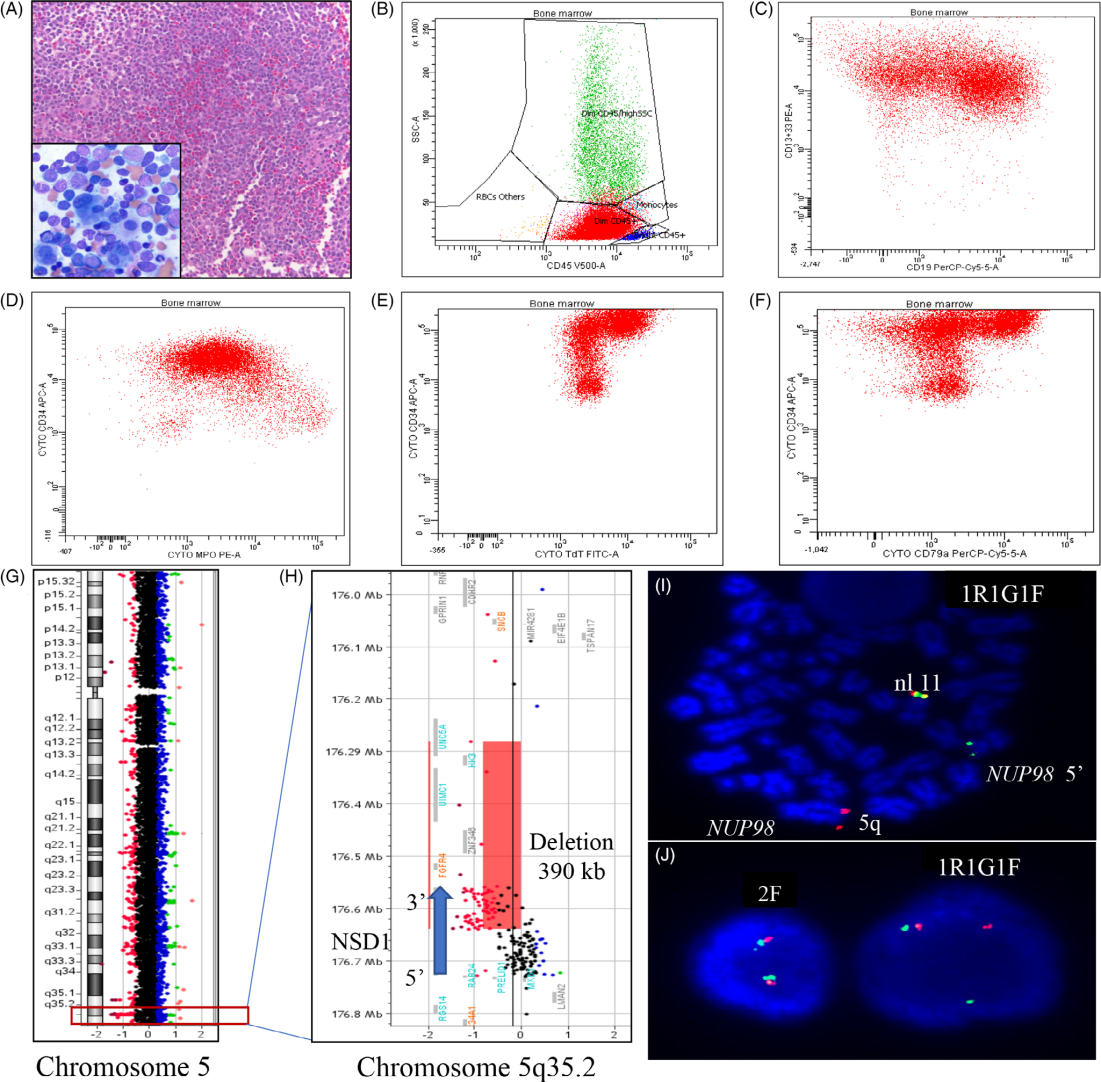
A, Histologic images shows hypercellular bone marrow effaced by leukemic infiltrate (Magnification ×100) (inset: marrow aspirate with many blasts, megakaryocytic and erythroid dysplasia [Magnification ×500]). Immunophenotypic analysis shows blasts which are: B, CD45 dim+ C, CD13/CD33+, partial CD19+ D, CD34+, dim MPO+ E, partial TdT+ F, partial CD79a+. G, Findings of Chromosome 5 microarray based comparative genomic hybridization (CGH) plot. H, Magnified view of a ~390 kb loss comprising the 3′ end of the *NSD1* gene (blue arrow), red rectangle indicates a loss in DNA copy number in the 5q35 region. I, FISH analysis using the *NUP98* breakapart probe is positive for the rearrangement on metaphase cells and J, interphase cells

The patient, a 13‐year‐old female, presented with constitutional symptoms and complete blood count (CBC) demonstrated a white blood cell (WBC) of 25.3 × 10^9^/L, hemoglobin of 5.7 g/dL, hematocrit of 16.5%, platelets of 63 × 10^9^/L, and 38% blasts with 58% blasts in the bone marrow (Figure 1A). Flow cytometric analysis (FCA) revealed a leukemic immunophenotype with expression of myeloid and lymphoid antigens: dim CD45+, CD34+, CD13+, CD33+, partial CD117+, partial CD7+, dim myeloperoxidase+, partial TdT+, partial CD19+, CD20‐, CD10‐, cytoplasmic CD3‐, HLA‐DR+, CD123+, CD38+. Additional analysis revealed two distinct blast populations, with one population expressing lymphoid antigens CD22+ and CD79a+ (Figure 1B‐F). This immunophenotype was consistent with B/myeloid (B/My) MPAL, as per current WHO 2017 guidelines.^7^ Chromosome analysis showed normal karyotype and fluorescence in situ hybridization (FISH) was negative for *KMT2A*, *ETV6*, *RUNX1*, *CBFB*, and *BCR/ABL* gene rearrangements, and microdeletions on 5q, 7q, and *PAX5*. Microarray revealed a deletion involving the 5′ portion of the *NSD1* gene, suggesting a *NUP98‐NSD1* rearrangement (Figure 1G,H). FISH analysis using the *NUP98* probe confirmed the cryptic t(5;11)(q35;p15.5) translocation resulting in the *NUP98‐NSD1* gene fusion in 90.8% of interphase cells (Figure 1I,J). Next generation sequencing (NGS) detected a *NRAS* p.Gly61Arg alteration.^8^

Given predominant myeloid features, and poor prognosis with a *NUP98‐NSD1* fusion, AML directed therapy was chosen with the goal of HSCT after remission.^9^ The patient's cytogenetic findings, and immunophenotypic responses are summarized in Table 1. The acute leukemia follow up panel has a level of threshold of detection of 0.1%, is based on a “different from normal” approach, and consists of seven tubes (marker combinations) which include 4‐color, 6‐color, and 8‐color tubes. The following antibodies are included in this panel: CD16, CD57, CD7, CD4, CD3, CD56, CD8, CD2, CD45, CD7, CD13, CD33, CD19, CD5, kappa, lambda, CD10, CD38, CD20, CD45, TdT, MPO, cytoplasmic CD3, CD34, CD22, CD79a, CD36, CD123, CD64, CD14, HLA‐DR, CD58. Despite morphologic and immunophenotypic remission after Induction II, an appropriate donor was not immediately available, so FLAG‐DaunoXome was used as a bridge to HSCT, which was complicated by sepsis and prolonged intubation. Bone marrow evaluation after this course showed no evidence of leukemic blasts by FCA, but persistent cytogenetic abnormality. Despite further delays from cholecystolithiasis requiring a cholecystectomy, repeat disease evaluation demonstrated no definite blast population by FCA, but 80.6% of cells were positive for the *NUP98‐NSD1* fusion.

**TABLE 1:** Bone marrow results by flow cytometry and cytogenetics throughout treatment

	% leukemic blasts by flow cytometry	FISH for % cells with NUP 98 rearrangement
Diagnosis	58%	90.80%
ADE 10+5+3 End of Induction I	11.30%	20.60%
HD‐Ara‐C Mitoxantrone End of Induction II	0%	negative (0.5%)
FLAG‐DaunoXome end of Cycle	0%	28.30%
Prior to RIC BMT Bu/Flu/ATG	No definite identified blast population by IHC	80.60%
Day +28 Bone Marrow	0%	3.70%
Day +60 Bone Marrow	24%	59.90%
3 months into Sirolimus/Azacitidine	23%	86.70%
After refractory to treatment	82%	97.30%

Abbreviations: ADE 10+5+3, cytarabine, daunorubicin, etoposide; FLAG‐DaunoXome, fludarabine, cytarabine, and granulocyte colony‐stimulating factor plus liposomal daunorubicin; RIC BMT Bu/Flu/ATG, RIC bone marrow transplant with busulfan, fludarabine, and anti‐thymocyte globulin.

Despite this persistent cytogenetic abnormality, the patient remained in flow cytometric remission, and received a 7/8 HLA mismatched unrelated allogenic HSCT 6 months after diagnosis. Reduced intensity conditioning (RIC) with busulfan, fludarabine, and anti‐thymocyte globulin (ATG) was chosen over myeloablative conditioning (MAC) given prior infectious complications. Graft‐vs‐host‐disease (GvHD) prophylaxis included tacrolimus and mycophenolate mofetil (MMF). She developed grade II skin acute GvHD and was treated with systemic and topical steroids. Day +60 bone marrow had 24% blasts by FCA with a similar immunophenotype to diagnosis, except near absence of CD19 expression. FISH revealed the *NUP98‐NSD1* fusion in ~60% of cells. Bone marrow chimerism analysis showed 59% donor cells.

Recent HSCT and acute GvHD precluded enrollment on clinical trials. Azacitidine and sirolimus were started with a subsequent decline in both the peripheral WBC count and blast count. Bone marrow evaluation after 3.5 months revealed 23% blasts by FCA and *NUP98‐NSD1* fusion in 87% of cells. Five months into therapy she developed a grade III septic joint, and both immunosuppressive agents were held. The patient experienced further disease progression 6 months from the start of relapse therapy and was transitioned to palliative care and died 2 months later.

## 3 DISCUSSION

This case highlights the challenges faced in choosing treatment options in MPAL patients with complex genomic features. The t(5;11)(q35;p15.5) translocation resulting in a *NUP98‐NSD1* fusion first described in a pediatric AML patient in 2001, is seen in 3% to 7% of pediatric AML cases, and confers a poor prognosis.^10^ Myeloid malignancies with NUP98 fusions, are often refractory to induction chemotherapy, and require consolidation with HSCT.^9^, ^11^ Functionally, the NUP98 protein belongs to the family of nuclear pore complex proteins that regulate nucleocytoplasmic transport of macromolecules.^12^ Additionally, NUP98 is involved in transcriptional regulation and it is hypothesized that aberrant *NUP98* fusions alter transcription of target genes leading to leukemogenesis.^13^ Schmoellerl et al recently showed that inhibiting expression of *NUP98* fusions, including *NUP98‐NSD1*, led to decreased disease burden in vivo, and transcriptional changes promoting normal hematopoiesis.^14^ Additional analysis of *NUP98* fusions, including *NUP98‐NSD1*, showed similar overexpression of target genes in oncogenesis, including *cyclin‐dependent kinase 5* (*CDK6*), a potential pharmacologic target.^14^

RAS pathway alterations are associated with hematologic malignancies, however this patient's *NRAS* p.Gly61Arg mutation is not common in blood cancers. It has been previously reported in an early T‐cell precursor (ETP) ALL patient with juvenile myelomonocytic leukemia features.^15^ *NRAS* encodes a member of the RAS family of GTPases that mediate transduction of growth signals. Constitutive activation of *NRAS* leads to activation of both the RAF/MEK/ERK and PI3K/AKT/mTOR pathways causing uncontrolled cell proliferation.^16^ Potential therapeutic options include sirolimus and everolimus, which bind directly to mammalian target of rapamycin (mTOR) complex 1.^17^

Recent genomic analysis of MPAL in pediatric and adult patients, which was reported after the treatment of this patient, highlight the genomic heterogeneity, and overall support more ALL‐directed therapy as the initial treatment choice. Takahashi et al analyzed the genomic landscape of adult MPAL and showed that both B/My and T/Myeloid (T/My) phenotypes share common genetic mutations associated with AML and ALL, but describe notable differences in somatic mutations and DNA methylation affecting gene expression.^6^ *NRAS* mutations were seen in 19.3% of cases, but none were in codon 61, and one patient with a *NUP98‐NSD1* fusion lacked a *NRAS* mutation.^6^ This group suggests that improved prognosis with ALL‐directed therapy may occur in patients with an ALL specific methylation profile.^6^ Furthermore, genomic analysis of 159 pediatric MPAL cases by Alexander et al showed that RAS pathway alterations were seen in 63% of B/My MPAL cases, with *NRAS* mutations contributing to a large proportion of these alterations.^4^ Among the 21 MPAL cases with *NRAS* mutations, two had a mutation in codon 61, including one with the specific p.Gly61Arg mutation, but lacked a concurrent *NUP98‐NSD1* fusion. This group also supported ALL‐directed therapy, as the genomic architecture of B/My MPAL was similar to ALL.^4^ Retrospectively, the patient's *NRAS* mutation, found to commonly occur in B/My MPAL in the above studies, likely contributed to the phenotype of her MPAL, and she potentially may have responded to ALL‐directed therapy. However, all known MPAL treatment data that supports upfront ALL‐based therapy is retrospective, and can be challenging to interpret given the changing definitions of this disease.^5^, ^18^, ^19^ The current Children's Oncology Group (COG) study AALL1732 (NCT03959085), is the very first clinical trial to enroll MPAL patients prospectively. This study is designed to treat all de novo MPAL patients with ALL‐directed therapy, but allows for patients with a poor treatment response to switch to AML chemotherapy followed by allogeneic HSCT.

Given the patient's high risk cytogenetic features, plan was to consolidate with HSCT with MAC, supported by a recent study showing superior outcomes in MPAL patients receiving MAC vs RIC^20^ Unfortunately, our patient's severe prior infectious toxicities precluded her from safely receiving MAC. Despite inferior survival with RIC, her rising cytogenetics with negative disease status by FCA demonstrated overall resistance of her disease to chemotherapy, so HSCT with RIC was pursued with goal of cure.

After relapse, tacrolimus was switched to sirolimus, in an attempt to target *NRAS* downstream by inhibiting mTOR.^17^ mTOR inhibitors in combination with additional agents have been studied in AML and other advanced cancers.^21^ An adult Phase Ib/II study of relapsed/refractory AML using azacitidine with everolimus was well‐tolerated with increased overall survival.^22^ The patient's response to combination therapy was suboptimal (Table 1) and was discontinued due to adverse effects secondary to immunosuppression.

In summary, we present this case to highlight the recent advances in understanding the genomics of MPAL. At the time of presentation, the cytogenetic features of the leukemia were suggestive of a phenotype most consistent with a myeloid leukemia. Although the patient initially responded to therapy, the toxicities associated with treatment made a long‐term remission difficult to achieve. Since this patient was treated, our understanding of the genetic landscape of MPAL has evolved, and informs treatment decisions to achieve better outcomes.

## CONFLICT OF INTEREST

The authors declare that there are no conflicts of interest.

## AUTHOR CONTRIBUTIONS

All authors had full access to the data in the study and take responsibility for the integrity of the data and the accuracy of the data analysis. *Conceptualization*, S.S.G., A.B.; *Methodology*, S.S.G.; *Formal Analysis*, S.S.G., S.A.Y., M.D., A.B.; *Writing ‐ Original Draft*, S.S.G., A.B.; *Writing ‐ Review & Editing*, S.S.G., S.S.R., S.A.Y., M.D., A.B.; *Visualization*, S.A.Y., M.D.; *Supervision*, A.B.; *Data Curation*, S.A.Y., M.D., A.B.; *Validation*, S.S.G., A.B.

## ETHICAL STATEMENT

Informed consent was obtained from a guardian given the patient was under 18 years of age at the time of death. This was published according to our institution's standards for case reports, and did not require IRB approval as it only includes one patient.

## Data Availability

The patient data used in the current case report are available from the corresponding author on reasonable request.
